# Teachers’ Perceptions of Bullying of Lesbian, Gay, Bisexual, Transgender, and Questioning (LGBTQ) Students in a Southwestern Pennsylvania Sample

**DOI:** 10.3390/bs5020247

**Published:** 2015-05-28

**Authors:** Jered B. Kolbert, Laura M. Crothers, Matthew J. Bundick, Daniel S. Wells, Julie Buzgon, Cassandra Berbary, Jordan Simpson, Katherine Senko

**Affiliations:** Department of Counseling, Psychology, and Special Education, Duquesne University, 409C Canevin Hall, 600 Forbes Avenue, Pittsburgh, Pennsylvania, 15282, USA; E-Mails: kolbertj@duq.edu (J.B.K.); bundickm@duq.edu (M.J.B.); wellsd1@duq.edu (D.S.W.); buzgonj@duq.edu (J.B.); berbaryc@duq.edu (C.B.); simpson7@duq.edu (J.S.); senkokathryn@gmail.com (K.S.)

**Keywords:** lesbian, gay, bisexual, transgender, bullying, teachers’ perceptions

## Abstract

This study was designed to ascertain teachers’ perceptions of bullying of Lesbian, Gay, Bisexual, Transgender, and Questioning (LGBTQ) youth. In a sample of 200 educators (61.0% female; 96.5% White) from a county in southwestern Pennsylvania, there was a significant positive relationship between the teachers’ perceptions of the supportiveness of school staff towards students regardless of sexual orientation and those teachers’ reports of the frequency of bullying victimization experienced by LGBTQ students. Teachers’ perceptions of a higher level of staff and student support was associated with higher reported frequencies of students’ use of derogatory language about LGBTQ individuals and various types of bullying of LGBTQ students. Teachers with a lesbian, gay, or bisexual orientation were found to rate the school staff and students as significantly less supportive of students regardless of their sexual orientation, gender identity, or gender expression in comparison to heterosexual teachers. Finally, teachers who either were unaware of or believed that their school lacked an anti-bullying policy reported significantly higher rates of physical bullying victimization of LGBTQ students when compared to the rates observed by teachers who reported knowledge of their schools’ anti-bullying policies.

## 1. Introduction

Although bullying has been described as a widespread problem among children and adolescents within schools (e.g., 28% of students reported experiencing bullying behaviors over the past academic year [1]), students who identify as Lesbian, Gay, Bisexual, or Transgender (LGBT) appear to be at an increased risk for the experience of peer bullying victimization. A recent, nationwide survey of over 7800 LGBT students indicated that 55.5% of respondents felt unsafe at school due to their sexual orientation, 74.1% had experienced verbal harassment, 36.2% had experienced physical harassment, and 49.0% had experienced cyberbullying [2]. Another study found that the LGBT students surveyed experienced such high levels of homophobic and transphobic bullying that the victimization became “normalized element(s) of their daily lives” [3] (p. 948). Unfortunately, researchers have noted that bullying victimization has the capacity to lead to a host of negative outcomes for adolescents, including depression, anxiety, and frequent school absences [4]. 

The adults in LGBT students’ lives, both parents and teachers, are likely to have first-hand knowledge regarding the experiences of sexually-diverse youth in terms of their day-to-day functioning. In particular, teachers may have a unique perspective of LGBT students’ peer relationships in the school setting, where children and adolescents spend much of their lives. Indeed, one of the theoretical perspectives that may be helpful to our understanding of bullying of LGBT youth is a social-ecological framework, in which bullying victimization is seen as complicated social exchanges among individuals, peer networks, and the broader social system [5]. Children’s environments are profoundly shaped by adults, who influence the social ecology in which youth develop [6]. Thus, for theoretical and pragmatic reasons, ascertaining educators’ perceptions of bullying of LGBT youth will be potentially helpful for the establishment of current epidemiological findings and intervention development. 

## 2. Students’ Reports to Educators and Educators’ Response to Bullying of LGBT Youth

Given the negative outcomes that are likely to accompany being bullied, perhaps even more disturbing are the rates at which LGBT youth report these incidents to school personnel. In a research investigation, of all the LGBT students who reported being victimized at their school, 56.7% of those students chose not to report the incident to school officials because they felt that interventions were unlikely to occur or that the situation could worsen if records were made of the event [2]. Due to the normative quality of these behaviors and interactions, the participants indicated that they would rarely report the bullying they experienced, which ranged from verbal intimidation to physical assaults [3].

Unfortunately, such fear is well placed, as 61.6% of LGBT students who reported bullying in the same study indicated that the school staff did not attempt to address the bullying [2]. Studies of LGBT youth have suggested that they do not feel support inside or outside of their school, and that when they did report homophobic bullying, many felt that school officials and/or the police did not view such incidents as seriously as other forms of bullying or aggression [3]. In one investigation, the majority of participants also felt that practitioners were not doing enough to educate youth about LGBT issues and how to address homophobic bullying [3]. Since it appears likely that LGBT students are likely to be specifically targeted among their peers within the school environment [2], and that teachers are frequently either unaware of these bullying behaviors or choose not to intervene, LGBT students appear to be particularly at risk for the negative effects of being bullied. 

## 3. Intervention in Bullying of LGBT Youth by Teachers

In a study surveying members of the National Education Association (NEA), Bradshaw and colleagues found that teachers and educational support professionals felt the least comfortable intervening with bullying regarding sexual orientation in comparison to any other special population (e.g., overweight, students with disabilities, *etc.* [7]). Similarly, a large survey distributed to Irish secondary schools found that while 87% of educators had witnessed LGBT bullying more than once, 41% of these educators had more difficulty addressing homophobic bullying than other types of bullying [8]. In another study, it was suggested that even when they are aware of the problem, educators may not address bullying behavior of sexually-diverse children and adolescents because of fear of discrimination, fear of job loss, the possibility of receiving unfavorable reactions from parents, students, and other staff members, their own prejudices, or failure to recognize bullying based on sexual orientation as a serious problem [8,9]. Indeed, in the NEA study, all groups assessed reported a need for more information on how best to intervene with sexual minority youth [7]. 

## 4. Protective Effects of Teacher Intervention in Bullying of LGBT Youth

Lending credence to this request, support by teachers and school staff has frequently been cited as a protective factor for sexual-minority youth. Research suggests that homophobic bullying is more prevalent in schools where educators remained uninvolved because of being unaware of bullying or unequipped with the appropriate training [10]. A study by Goodenow, Szalacha, and Westheimer found that sexual minority youth who believed that there was no adult in the school to whom they could talk about a problem were more likely than others to have been threatened at school and to have made multiple suicide attempts in the previous year [11]. 

Conversely, support from staff was a significant protective factor against suicide attempts, even when victimization was taken into account [11]. Adding support to this finding, McGuire, Anderson, Toomey, and Russell found that a sample of 59 transgender students reported that they felt greater levels of school safety and connectedness when educators and administrators took active roles in bullying prevention [12], while students who experience homophobic victimization tend to have stronger school commitment when they can identify an educator who they believe is caring [13]. 

Some efforts have focused upon developing schools to provide more supportive environments for sexual minority youth. These approaches have included training for school staff to increase sensitivity and awareness, adding materials related to gay and lesbian issues in the curriculum, and attempting systemic change of the school culture to increase acceptance of diversity [11]. There is evidence from research suggesting that some of these approaches are effective. In schools where health teachers reported delivering HIV education they believed to be appropriate for LGBT youth, sexual minority students reported lower rates of many health risk behaviors, including high-risk sexual behaviors, skipping school due to fear, and planning suicide attempts. Likewise, a significant association has been found between staff training regarding sexual diversity and an improved school climate for sexual minority students; however, most efforts to improve the school environment for sexual minority youth have not been carefully evaluated [11].

## 5. Inadequacy of Current Intervention

Although the research literature indicates that LGBT youth feel greater support when they receive teacher encouragement and experience a positive school climate, existing school interventions may not be sufficient to assuage the effects of being bullied. A national survey was distributed to teachers to gauge their perceptions and practices regarding school bullying prevention, and revealed that 86.3% of respondents indicated that their only bullying intervention included “serious talks” with both parties of the incident [14], despite fewer than one in five educators perceiving that bullying was not a problem within their classrooms [14]. 

The existing responses from schools and educators regarding bullying of LGBT youth are encouraging, yet insufficient. In an attempt to identify the impediments to more nurturing and effective educators’ responses and school-based interventions regarding the bullying of LGBT students, the current study aims to further understand educators’ perceptions of the support given to LGBT students within schools. This study addresses two overarching research questions, including: (1) How do teachers perceive the level of support provided to sexual minority youth within their schools, and how does the perceived support relate to the reported levels of bullying of LGBT students? and (2) Does the existence of explicit bullying policies and programs impact teachers’ perceptions of the bullying of LGBT students in schools? Moreover, this research study was undertaken in an effort to expand the literature base in documenting educators’ perspectives of the experience of LGBT students in schools and potentially using such information to better develop interventions and policies to protect LGBT students from bullying.

## 6. Methods

### 6.1. Participants

In order to assess teachers’ perceptions of lesbian, gay, bisexual, transgender and questioning (LGBTQ) students’ bullying experiences as well as teachers’ knowledge of anti-bullying policies, 3652 middle, junior high, and secondary school educators employed in 42 different school districts in a single southwestern Pennsylvania county were contacted via email and invited to participate in the current study. Teachers’ email addresses were obtained through a review of each district’s publically available website. Teachers who were described as working primarily with students in grades eight through twelve and whose email address was publically available was invited to participate. The email invitation provided a description of the study, and links to the consent form and survey. 

After a two month data gathering period, which consisted of an initial invitation to participate and one emailed reminder, 217 different educators completed at least a portion of the distributed survey, representing a response rate of 5.94%. This response rate is lower than expected given recent research documenting the effectiveness of web-based surveys in attracting participants. For example, one meta-analysis of web-based survey response rates cited 45 different studies in which response rates ranged from 11.13% to 82.13%, with an average response rate of 32.65% [15]. 

Other studies that have experimentally examined the effects on response rates when participants were recruited using different methods have shown that web-based surveys consistently produce the lowest response rates, while these rates are still well above what was obtained in this investigation (e.g., 20.4% [16] and 17.1% [17]. This tendency for web-based surveys to yield the lowest response rates appears to have persisted as internet literacy has increased; participants seem to still respond best to recruitment methods that employ several tactics, including postal mail [18]. 

McPeake, Bateson, and O’Neill make several suggestions to researchers hoping to maximize their response rate on internet-based surveys [19]. These suggestions include personalizing email invitations, attempting to limit the number of undeliverable invitations through careful recording of addresses, sending at least two reminder emails, and embedding the link to the survey in the invitation email. While the current investigation followed some of these suggestions, the nature of the investigation limited the applicability of other tactics. For example, the current research team was able to embed the link to the survey in the invitation email and also limited the number of undeliverable emails through careful recording of email addresses. However, only one reminder email was sent to possible participants and, due to the need for anonymity of the sample, email invitations were not personalized. Other factors that may have negatively impacted the response rate of the current investigation include the sensitivity of the topic discussed, the tendency for email invitations to surveys being considered “spam” by possible participants [18], school district firewalls that may have restricted teachers’ ability to access the survey, the length of the survey, and the lack of any incentives to complete the survey. 

Of the 217 educators who responded, only 192 subjects completed every item of the distributed survey. In order to minimize the effects of any missing data and maximize the number of responses, a data imputation procedure was completed and the expectation maximization data imputation method was chosen. Expectation maximization is an empirically validated statistical process that uses regression methods based on observed data to calculate the best estimate for pieces of missing data. The analysis then measures the resulting impact of the data’s addition to ensure that that individual data estimates do not cause significant changes in the overall parameter estimates [20]. After completing the expectation maximization imputation procedure, 201 complete responses were gathered. One participating teacher reported working primarily in an elementary school. Due to the current study’s focus on middle and high school teachers, this respondent was dropped from the subsequent analyses. This resulted in a final sample of 200 teachers which were used in the following analyses.

### 6.2. Measures

At present, there is not an empirically-validated scale that measures the constructs investigated in the current study. For this reason, the researchers developed a questionnaire that was tailored to the particular needs of the study. This scale was developed by two doctoral-level researchers conversant in the topic of bullying and LGBTQ issues, along with a research team of three master’s-level students. Information was also gathered from local and national LGBTQ advocacy groups, as well as school-based professionals in order to better understand the current experiences of the study’s target populations. The developed scale was used to evaluate educators’ perceptions regarding the bullying of LGBTQ students. The 35-question survey included demographic questions pertaining to the participants’ gender, age, race, school, religion, political affiliation, and sexual orientation. The survey also included questions regarding educators’ perception of school support for students, educators’ exposure to the LGBTQ community, and educators’ perception of the schools’ policies regarding bullying. 

After completing the initial demographic questions, most items required participants to respond to questions using a five-point Likert scale. The majority of the Likert scale response options ranged from “Never” to “Always”. Other response options included Likert scales ranging from “Much Less” to “Much More” or “Strongly Disagree” to “Strongly Agree”, depending upon the wording of the question. Participating educators also responded to multiple-choice questions regarding their students’ grades, where they most often witness bullying, to whom they feel comfortable reporting bullying, and how many students and teachers describe themselves as LGBTQ. Participants were given the option to write in their own responses to certain questions (e.g., the places they have witnessed or been made aware of bullying and to which school personnel they feel comfortable reporting bullying in the school). 

As the survey was developed specifically for the current investigation, developed items were able to be worded in a precise manner relevant to the study. Based on the researchers’ knowledge of the relevant literature base, practical experiences, and input from other valuable stakeholders, the research team was able to develop single item scales which demonstrate strong face validity in measuring the intended constructs. Due to the clear relationship between the survey items and constructs of interest, the developed survey is thought to provide reliable estimates of participants’ true thoughts, perceptions, and behaviors. 

After completion of the survey, four items were combined in order to form an Overall Bullying scale. These four items explicitly asked participants to report their perceptions of student experiences of verbal bullying, physical bullying, relational bullying, and cyberbullying. Once combined, the scale provides an overall estimate of teachers’ perceptions of the rate of LGBTQ student bullying victimization. As stated, the developed scale consisted of four items and was found to have a strong internal consistency (Cronbach’s alpha = 0.78). 

### 6.3. Procedures

Prior to the initiation of the investigation, the research team sought and received approval from the Institutional Review Board (IRB) of the corresponding author’s home university. Once approval was granted, teachers were invited to participate in the study through an e-mailed invitation. In order to ensure adequate participation, teachers were given the option to have their names entered in a drawing for one of three prizes (e.g., $50, $75, and $100 Visa online gift cards). In order to participate in the study, teachers clicked on the link to the survey provided in the e-mailed invitation. The survey was developed and distributed through the SurveyMonkey online survey website. The SurveyMonkey software allows researchers to construct and distribute unique surveys and collect responses through the construction of a password protected database without recording respondents’ personal information, therefore ensuring anonymity. Participants provided consent by choosing to continue to the hypertext links transporting them to complete the questions that were posed in this study. Participants had the option to withdraw during or at the completion of the survey by selecting an option that prevented their responses from being entered in the dataset. 

## 7. Results

### 7.1. Descriptive Statistics

This sample of the 200 respondents was comprised of 61% females, and the majority (96.5%) reported their race/ethnicity as White (see Table 1). Based on the anonymity requirement of data collection procedure, teachers did not report the school district where he or she was employed, and, therefore, specific comparisons between the gathered sample and population could not be made. In order to estimate the similarities between the gathered sample and the population, the Pennsylvania Department of Education (PDE) and United States Census data were consulted. Pennsylvania Department of Education statistics indicate that in the county surveyed, 71% of teachers are female [21] while Census data indicated that 81.3% of the county reported their race/ethnicity as White [22]. This data suggest that the current sample is similar to the overall population with some underrepresentation of female teachers and overrepresentation of White individuals. The average age of the respondents was found to be 40.6 years (*SD* = 8.16). Sixty-six point five of respondents reported working primarily in a high school setting, while 33.5% reported working in a middle school. Finally, 14 (7%) reported that they identified as having a sexual orientation other than “Straight”, with 10 (5%) identifying as “Gay/Lesbian” and 4 (2%) identifying as “Bisexual.” One participating teacher reported working primarily in an elementary school. Due to the current study’s focus on middle and high school teachers, this respondent was dropped from the subsequent analyses. This resulted in a final sample of 200 teachers which were used in the following analyses.

**Table 1:** Descriptive statistics of sample.

Category	Number (N = 201)	Percentage
Gender		
Male	78	38.8%
Female	123	61.0%
Race/Ethnicity		
White	194	96.5%
Black	2	1%
Asian	0	0%
Hispanic	1	0.5%
Native American	0	0%
Biracial	2	1%
Sexual Orientation		
Heterosexual	186	93%
Gay/Lesbian	10	5%
Bisexual	4	2%
School Level Taught		
High School	133	66.5%
Middle School	67	33.5%

### 7.2. Educators’ Perceptions of Supportiveness

When educators were asked about the frequency with which they perceived LGBTQ students being supported by members of the school staff, the majority of teachers rated their schools positively and stated that their schools either “Always” (51.5%) or “Frequently” (33.0%) support all students regardless of their sexual orientation, gender identity, or gender expression. Teachers tended to report that students in the schools in which they work are less commonly supportive of their peers (in comparison to teachers) regardless of their sexual orientation, gender identity, or gender expression, as the responding educators rated their students as only being “Frequently” (57.0%) or “Sometimes” (32.5%) supportive of their peers. Despite these differences, teachers, in general, seem to perceive schools as being supportive of students regardless of students’ sexual orientation, gender identity, or gender expression. 

However, teachers who reported school support (both from educators and peers) for LGBTQ students were also more likely to perceive LGBTQ students as experiencing a higher level of victimization than non-LGBTQ students. Pearson correlations (see Table 2) indicate a significant positive relationship between the teachers’ perceptions of the supportiveness of school staff towards students regardless of sexual orientation and those teachers’ reports of the frequency of all bullying victimization experienced by LGBTQ students. Teachers’ perceptions of a higher level of staff supportiveness was associated with higher reported frequencies of students’ use of derogatory language about LGBTQ individuals (*r* = 0.14, *p* = 0.045), teachers’ use of derogatory language about LGBTQ individuals (*r* = 0.19, *p* = 0.008), verbal bullying (*r* = 0.22, *p* = 0.002), physical bullying (*r* = 0.15, *p* = 0.03), relational bullying (*r* = 0.23, *p* = 0.001), sexual harassment (*r* = 0.20, *p* = 0.004), and cyberbullying (*r* = 0.14, *p* = 0.046), as well as increased frequencies of an overall bullying experience (*r* = 0.24, *p* = 0.001) for LGBTQ students.

Similarly, Pearson correlations (see Table 2) suggested that teachers’ perceptions of higher levels of peer support was related to higher reported frequencies of students’ use of derogatory language toward LGBTQ individuals (*r* = 0.27, *p* < 0.001), physical bullying (*r* = 0.21, *p* = 0.003), relational bullying (*r* = 0.23, *p* = 0.001), sexual harassment (*r* = 0.24, *p* < 0.001), and cyberbullying (*r* = 0.15, *p* = 0.038), as well as higher reports of the overall frequency of bullying victimization toward LGBTQ youth (*r* = 0.23, *p* = 0.001). Teachers’ perceptions of students’ peer support were not significantly related to the frequency of the teachers’ use of derogatory language or the experience of verbal bullying toward or of LGBTQ students. While these results may be associated with increased awareness being related to increased reports as discussed above, further analysis suggests that some of the teachers’ perceptions may be inaccurate. 

**Table 2:** Pearson Correlations between Support and Bullying Variables.

	School Personnel Support	Student Support	Verbal Bullying	Physical Bullying	Relational Bullying	Sexual Harassment	Cyber Bullying	Student Derogatory Language	Teacher Derogatory Language	Overall Bullying
School Personnel Support	1	0.42 **	0.21 **	0.15 *	0.23 **	0.20 **	0.14 *	0.14 *	0.19 **	0.24 **
Student Support	0.42 **	1	0.13	0.21 **	0.23 **	0.24 **	0.15 *	0.27 **	0.13	0.23 **
Verbal Bullying	0.22 **	0.13	1	0.45 **	0.62 **	0.49 **	0.41 **	0.39 **	0.25 **	0.80 **
Physical Bullying	0.15 *	0.21 **	0.45 **	1	0.38 **	0.56 **	0.38 **	0.20 **	0.09	0.68 **
Relational Bullying	0.23 **	0.23 **	0.62 **	0.38 **	1	0.52 **	0.56 **	0.42 **	0.14	0.83 **
Sexual Harassment	0.20 **	0.24 **	0.49 **	0.56 **	0.52 **	1	0.44 **	0.36 **	0.17 *	0.64 **
Cyber Bullying	0.14 *	0.15 *	0.41 **	0.38 **	0.56 **	0.44 **	1	0.16 **	0.01	0.79 **
Student Derogatory Language	0.14 *	0.27 **	0.39 **	0.20 **	0.42 **	0.36 **	0.16*	1	0.41 **	0.38 **
Teacher Derogatory Language	0.19 **	0.13	0.25 **	0.09	0.14	0.17 *	0.01	0.41 **	1	0.15 *
Overall Bullying	0.24 **	0.23 **	0.79 **	0.68 **	0.83 **	0.64 **	0.79 **	0.38 **	0.15 *	1

* significant at 0.05; ** significant at 0.01.

When the responses of those teachers who report a lesbian, gay, or bisexual orientation are compared with those responses of teachers who identify as heterosexual, the teachers with a gay, lesbian, or bisexual orientation were found to rate the school staff in the buildings in which they work as significantly less supportive of students regardless of the students’ sexual orientation, gender identity, or gender expression, *t* (198) = 4.95, *p* < 0.001) with an effect size (Cohen’s d) of 1.49. Equality of sample variances was assured through Levene’s test, which produced a non-significant result (*F* = 0.111, *p* = 0.74). Furthermore, lesbian, gay, or bisexual teachers also rated the students in their school as significantly less supportive of their peers regardless of their peers’ sexual orientation, gender identity, or gender expression, *t* (198) = 2.08, *p* = 0.039) with an effect size (Cohen’s d) of .65, in comparison to heterosexual teachers. Again, Levene’s test indicates that the sample variances are not significantly different (*F* = 0.286, *p* = 0.594). These findings may suggest that those teachers who are most aware of the experiences of LGBTQ students, based upon their own adolescent experiences (e.g., lesbian, gay, or bisexual teachers), perceive the school environment as significantly less supportive than teachers who may be less aware. Interestingly, however, lesbian, gay, or bisexual teachers do not perceive a higher rate of bullying of LGBTQ students, *t* (198) = 1.26, *p* = 0.210) with an effect size (Cohen’s d) of 0.38, as compared to their heterosexual colleagues. Sample variances, again, are not found to be significantly different by Levene’s test (*F* = 0.021, *p* = 0.886).

### 7.3. Effects of Teachers’ Knowledge of School Anti-Bullying Policies

As discussed, student bullying has been a significant focus of educational policy and legislative groups who have attempted to enact guidelines and programs to limit the rates of bullying. In fact, Pennsylvania state law requires every school district to publish an anti-bullying statement or policy describing that student bullying is not permitted in the district [23]. A recent report conducted in tandem with the current study found that each of the 42 districts from which teachers were recruited for the current study were in compliance with this state law; meaning that each of the schools where the surveyed teachers were employed had a policy that explicitly disallowed student bullying [24]. 

However, these reports were found to vary in their level of specificity, especially in regards to their treatment of sexual minority students. It was found that most districts’ policies (e.g., 93%) regarding bullying did not identify particular populations as being in need of protection. While educators who responded on the online survey were not asked to report the district in which they were employed and, therefore, their responses could not be matched to a specific district’s anti-bullying policy, the overall rates of policy existence and specific mention of LGBTQ student concerns will provide qualitative comparisons of teachers’ responses. 

When educators were simply asked whether their school district had an explicit anti-bullying policy, 29 respondents (14.5%) indicated that their district did not have such a policy or that they were unaware if such a policy existed, even though each school district from which the teachers were recruited for this study did have such a policy. Furthermore, when educators were asked if the anti-bullying policy that was in place at their district contained language specific to LGBTQ students, 51 respondents (25.4%) reported that their district’s policy did contain such specific language, which is somewhat surprising given that only 7% of the districts were found to have policies that actually contained such statements. These findings suggest that, even if school districts are attempting to affect the rates of bullying victimization through the implementation of anti-bullying policies, those educators responsible for implementing the policies may not be aware of both of the policies’ existence and/or the information discussed within those policies. 

This lack of awareness also appears to be related to teachers’ perceptions of whether their schools are doing enough to prevent student bullying victimization. Those teachers who did not believe or were unaware that their school district had an anti-bullying policy rated their school significantly lower in terms of whether or not the school was doing enough to prevent bullying in general, *t* (198) = 3.86, *p* < 0.001) with an effect size (Cohen’s d) of 0.73. The variance between the two measured groups was not found to be significantly different by Levene’s test (*F* = 0.79, *p* = 0.375). The perception of schools not doing enough persists when educators were asked to consider the treatment of LGBTQ students. Those teachers who did not believe or who were unaware that their school’s anti-bullying policy contained LGBTQ-specific language rated their schools significantly lower in terms of doing enough to prevent bullying of LGBTQ students, *t* (198) = 4.3, *p* < 0.001) with an effect size (Cohen’s d) of 0.72. Again, groups were shown to demonstrate equality of variance by Levene’s test (*F* = 0.85, *p* = 0.358). 

Finally, given that teachers who are not fully aware of the presence or content of the established anti-bullying policies perceive that their schools are not doing enough to protect all students in general, and LGBTQ students specifically, from bullying victimization, analyses were completed to determine if these teachers reported being made aware of bullying experiences by students based upon their sexual orientation or gender expression. Through a one-way Analysis of Variance (ANOVA), it was determined that teachers who either were unaware of or believed that their school lacked an anti-bullying policy reported significantly higher rates of physical bullying victimization of LGBTQ students when compared to the rates observed by teachers who reported knowledge of their schools’ anti-bullying policies, *F* (1198) = 7.89, *p* = 0.005 (see Table 3). However, knowledge of schools’ anti-bullying policies was not significantly related to differing rates of any other type of peer aggression toward LGBTQ youth. Similarly, knowledge of the content of schools’ anti-bullying regarding the presence of specific sexual orientation or gender expression protections was also not significantly related to the rates at which teachers were made aware of student aggression (see Table 4). 

**Table 3:** ANOVA Results of Teachers Who Were and Were Not Aware of Schools’ Anti-Bullying Policies.

		Sum of Squares	Degrees of Freedom	Mean Square	F	*p*-value
Verbal Bullying	Between Groups	0.59	1	0.59	9.27	0.34
	Within Groups	125.00	198	0.63		
	Total	125.58				
Physical Bullying	Between Groups	3.40	1	3.40	7.89	0.00 **
	Within Groups	85.32	198	0.43		
	Total	88.72	199			
Relational Bullying	Between Groups	1.55	1	1.55	2.47	0.12
	Within Groups	124.45	198	0.63		
	Total	126.00	199			
Sexual Harassment	Between Groups	0.00	1	0.00	0.01	0.94
	Within Groups	98.55	198	0.50		
	Total	998.55	199			
Cyberbullying	Between Groups	0.30	1	0.30	0.35	0.56
	Within Groups	169.69	198	0.86		
	Total	169.96	199			
Overall Bullying	Between Groups	10.95	1	10.95	1.80	0.18
	Within Groups	1208.17	198	6.10		
	Total	1219.12	199			

* significant at 0.05; ** significant at 0.01.

**Table 4:** ANOVA Results of Teachers Who Were and Were Not Aware of Specific Language Related to Specific Sexual Orientation and Gender Expression Language.

		Sum of Squares	Degrees of Freedom	Mean Square	F	*p*-value
Verbal Bullying	Between Groups	1.44	1	1.44	2.92	0.13
	Within Groups	124.14	198	0.63		
	Total	125.58				
Physical Bullying	Between Groups	0.05	1	0.05	0.12	0.73
	Within Groups	88.67	198	0.45		
	Total	88.72	199			
Relational Bullying	Between Groups	0.71	1	0.71	1.13	0.29
	Within Groups	125.29	198	0.63		
	Total	126.00	199			
Sexual Harassment	Between Groups	0.03	1	0.03	0.06	0.80
	Within Groups	98.52	198	0.50		
	Total	99	199			
Cyber Bullying	Between Groups	0.32	1	0.32	0.38	0.54
	Within Groups	169.66	198	0.86		
	Total	169.98	199			
Total Bullying	Between Groups	1.33	1	1.33	0.22	0.64
	Within Groups	1217.79	198	6.15		
	Total	1219.12	199			

* significant at 0.05; ** significant at 0.01.

## 8. Discussion 

In the first research question of the study, how do teachers perceive the level of support provided to sexual minority students within their schools, there are a number of possible interpretations regarding the apparent contradictory finding that teachers’ perceptions of staff and students in their school being supportive of LGBTQ students was positively associated with bullying of LGBTQ students and the use of derogatory language by both staff and students. While lesbian, gay, or bisexual teachers reported lower levels of staff and student supportiveness for LGBTQ students than did heterosexual teachers, LGB teachers may have a more accurate perception of the supportiveness of staff and students than do their heterosexual colleagues. It may be that staff and student support is heightened in an environment that is characterized by the use of derogatory language toward and bullying of LGBTQ students. 

In the first research question of the study, experimenters also wished to learn how the perceived support related to the reported levels of bullying of LGBTQ students. In response to this question, results suggested that heterosexual teachers may wish to see the staff and students in their school as being as equally supportive of LGBTQ and heterosexual students who are experiencing bullying victimization, when that is unlikely. Indeed, the issue of sexual orientation may constitute a source of emotional sensitivity for non-LGB teachers, who desire to perceive the school environment as being equally supportive for both LGBTQ and heterosexual students in deference to political sensibilities. Heterosexual teachers may not recognize that the school environment is not as supportive for LGBTQ students as it is for heterosexual students, because this would imply that they would need to engage in action to rectify this source of unequal treatment. Support for this argument lies in the fact that studies have found that teachers feel unprepared to help LGBTQ students [3]. 

An alternative argument regarding the apparent contradiction in the results may be that heterosexual teachers’ perceptions of the general school environment are, indeed, accurate, and their reports regarding the rates of bullying victimization are in reference to a minority population of students. In other words, the school environment is generally supportive of LGBTQ students; however, some staff and students in the school are particularly hostile to LGBTQ youth and engage in derogatory language/bullying. This possibility suggests that such teachers see the school as being politically polarized, with most of the staff and students being in support of LGBTQ students, in opposition to the minority of the staff and students. While the results of this study were not necessarily supportive of increased bullying awareness resulting in a greater likelihood of LGBTQ students being victimized in comparison to their heterosexual peers, it may be that increased awareness of the issue of bullying results in a polarization of the school community. Some teachers and students may react negatively to the call for the protections of students, particularly LGBTQ youth, while teachers supportive of LGBTQ students remain stalwart in their beliefs.

In this study, there were some interesting findings in comparing the responses of LGB and heterosexual teachers. Pointedly, LGB teachers perceived the school staff and students as being less supportive of bullying victims, but did not identify differences between educators’ and students’ support of LGBTQ *vs.* non-LGBTQ students. An argument may be constructed that if LGB teachers’ perceptions are more accurate than heterosexual teachers, it is because sexual-minority teachers have less of a need to say things are going well. In other words, their identification as a member of a minority population, one that has been traditionally victimized, affords them the insight that the conditions are not as good as they seem, although the school climate in regards to bullying victimization is not necessarily different for LGBTQ students and their heterosexual peers.

In the second research question of this study, does the existence of explicit bullying policies and programs impact teachers’ perceptions of the bullying of LGBT students in schools?, the results suggest several straightforward implications for bullying prevention. Teachers who did not believe in or who were unaware of the existence of their school’s anti-bullying policy were less likely to report that their school was doing enough to prevent bullying. This implies that school leaders need to either increase the frequency or methods in which they communicate anti-bullying policies to the educational staff. A recent meta-analysis regarding the effectiveness of bullying prevention programs found that there is a dosage effect, meaning that schools that have a greater number of bullying prevention activities and employ them more frequently are more likely to reduce bullying [25]. Teachers who did not believe in or were unaware of their school’s anti-bullying policy’s identification of LGBTQ students as warranting protection were significantly less likely to report that their school was doing enough to prevent bullying of LGBTQ students. A possible interpretation of this finding is that teachers may be supportive of efforts by states or schools to identify LGBTQ individuals as a group in need of protection from bullying. 

### 8.1. Future Research

Several of the findings of this study warrant further attention. Specifically, an ethnographic, qualitative study may be helpful in studying the mutual impact of the establishment of school policies to protect victims of bullying and teachers’ perceptions and response to such policies. Furthermore, such a qualitative investigation could examine students’ perceptions regarding teachers’ reactions and responses to the establishment of bullying policies. Mulcahy, Dalton, Kolbert, and Crothers found that in pursuing informal mentors/allies among teachers, LGBT students would look for subtle clues about teachers’ beliefs about issues of gender and religion in order to assess their potential views on differences in sexual orientation [26]. Likewise, it is possible that students who are generally unsupportive of policies or programs to provide protection to traditionally oppressed groups will seek subtle support from teachers who are like-minded. 

### 8.2. Summary

When educators and school staff are not adequately trained to be allies to LGBTQ students, they are not prepared to respond to their needs. This lack of response likely communicates an implicit message of approval of the harassment of LGBTQ youth and contributions to an unsafe environment for such students. Of importance, for LGBTQ students, the presence of supportive adults can help to create a welcoming and safe environment in which LGBTQ students can learn, achieve, and develop. This study provides a contribution to the understanding of the variables that relate to teachers’ responses to bullying of LGBTQ youth, which also may help to explain the rates of bullying of these students. 
